# Risk factors for tuberculosis in older children and adolescents: a matched case–control study in Recife, Brazil

**DOI:** 10.1186/s12982-014-0020-5

**Published:** 2014-12-30

**Authors:** Hilary Stevens, Ricardo AA Ximenes, Odimariles MS Dantas, Laura C Rodrigues

**Affiliations:** Department of Infectious Disease Epidemiology, Faculty of Epidemiology and Population Health, London School of Hygiene and Tropical Medicine, Keppel Street, London, WC1E 7HT UK; Department Medicina Tropical, Universidade Federal do Pernambuco, Bl. A do Hospital das Clinicas. Av. Prof. Moraes Rego s/n, Cidade Universitária, Recife, PE 50670-420 Brazil; Department Medicina Interna, Universidade de Pernambuco, Recife, Brazil; Department de Saúde Materno Infantil, Universidade Federal do Pernambuco, Recife, Brazil

**Keywords:** Tuberculosis, Adolescents, Risk factors, Household contacts, Socio-economic factors, Smoking

## Abstract

**Background:**

Tuberculosis is a major disease worldwide and most research focus on risk factors for adults, although there is a marked adolescent peak in incidence. The objective of this study was to identify risk factors for tuberculosis in children aged 7 to 19.

**Methods:**

A case control study matched by age with 169 cases and 477 controls. The study population consisted of adolescents and older children from Recife, Brazil. Cases were individuals diagnosed with tuberculosis in the control programme and controls were selected in the neighborhood of cases. Conditional logistic regression was used to identify risk factors.

**Results:**

Cigarette smoking increased by 50% the risk of tuberculosis but that this was not statistically significant (OR = 1.6). Other risk factors were sleeping in the same house as a case of tuberculosis (OR = 31.6), living in a house with no piped water (OR = 7.7) (probably as a proxy for bad living conditions), illiteracy (OR = 3.7) and male sex (OR = 1.8). The increase in risk with living in houses with no piped water was much more marked in males. The proportion of cases of tuberculosis attributed to contact with someone with TB was 38% and to illiteracy, lack of piped water and smoking, 20%.

**Conclusion:**

Household contact with tuberculosis, social factors and male sex play the biggest role in determining risk of TB disease among children and adolescents in the study. We recommend further research on the relationship of cigarette smoking on tuberculosis in adolescents, and on whether the sex differentials are more marked in bad living conditions. Separate studies should be conducted in older children and in adolescents.

## Background

Tuberculosis (TB) causes 1.3 million deaths per year worlwide [1]. It is estimated that, in 2013, 9.0 million people developed TB and that the global number of TB case notifications among children was 300 000 [2]. Tuberculosis infection is transmitted from person to person; likelihood of transmission increases with close and sustained exposure to a person with active pulmonary disease [3]. Only 10% of infected individuals develop disease [3].

Because not all infections lead to disease, risk factors can increase the risk of tuberculosis by increasing risk of acquiring infection or increasing risk of developing clinical disease. There is evidence of an increased risk of tuberculosis for immigrants from areas of high risk [4,5], males [6,7], those with family history of TB [8], and those working in health care settings [9]. Poverty and crowding have also been implicated. It is harder to study separately risk of developing disease once infection is acquired but co-infection with HIV and with parasites, low vitamin D [10], and smoking have been suggested [11-13], probably through suppression of the host immune response. Smoking increases susceptibility to TB through reducing ciliary activity and mucus production by goblet cells, the pulmonary system’s first line of defense [14]. Specific risk factors vary by region [15] and it is very likely that are different in children and adolescents from adults.

We report results of a study of socioeconomic and biological risk factors for tuberculosis in children aged 7 to 19 in the city of Recife, Brazil. Incidence of tuberculosis in Recife is relatively high in this age group: the average notification rate of tuberculosis (for 2001 to 2007) was, for children aged 7 to 14, 17.3 per 100 000 persons per year and for those adolescents aged 15 to 19, 69.5 per 100 000 persons per year [16].

## Methods

The data used in this study was from a case–control study of the protection against tuberculosis provided by a second dose of bacilli Calmette-Guerin (BCG) vaccine; this has been published [17]. We now report on the analysis of social factors for tuberculosis, using the same cases and control. Methods are reported elsewhere [17] but, in brief, the study population consisted of children aged 7–19 years residing in the metropolitan region of Recife. Cases were selected from subjects with newly diagnosed TB by the National TB programme (NTP), regardless of clinical form. All tuberculosis cases in Brazil are treated by the NTP, totally free of charge. The diagnosis of TB was reviewed by two independent experts who, based on clinical, epidemiological, laboratory and chest X-ray information, from the health unit registry and from the patient’s medical record before the expected end of treatment verified the diagnosis. Because the original study aimed to evaluate the effect of BCG revaccination, children with no BCG scar or more than 2 scars (presumed to result from neonatal BCG vaccination) were excluded. Only 3.9% of potential cases and 7.1% of potential controls were excluded for these reasons. Cases were also excluded if both reviewers judged them not to be TB or if there was a change in diagnosis during the treatment period. Only 3 cases were excluded for these reasons.

Matched neighborhood controls were selected based on the address of the case. The first three addresses were ignored and the next houses visited sequentially until three children of the same age group of the case were identified and recruited. The three controls had to live at three different addresses. When more than one child/adolescent was eligible in the same household the one whose month of birth was closer to that of the case would be selected. When the child/adolescent was not home an appointment to another visit was made. Controls were matched within the age groups 7–9, 10–14 and 15–19 years. The sole exclusion criterion for controls was a diagnosis of TB (none was excluded).

Interviews using a standardized questionnaire were conducted with cases and controls, if aged 15 years or more or with their parents/guardians if below that age. Cases were interviewed at the health unit providing TB treatment, while controls were interviewed in their homes. All of the information in the dataset was derived from the questionnaire, which included a total of 216 variables, from which the 12 variables (focused on biological, contact history and socioeconomic risk factors) analyzed here were constructed. Cut of points were used for all variables based on the literature or on examination of the data. Variables are presented here as characteristics of the individual, the head of the household or the residence.

### Individual characteristics

**Age: grouped in** three levels (7–19). **Alcohol:** as a binary variable, drinkers and no drinkers (no drinkers also included not known and not applicable, a large group given that approximately 80% of subjects were aged under 15). **Smoking:** as a binary variable: ever smoked and never smoked. **Contact with tuberculosis:** three levels: no contact, contact with someone who had tuberculosis sleeping in the same house or contact who did not sleep in the same house. **Relationship to head of household:** as a binary variable including those who were first degree relatives versus those who were either second degree relatives or not related at all. **Born in this district, or not:** proxy for migration. **Literacy** as a binary variable.

### Head of household characteristics

(i) whether or not the head of household was paid for work in the previous week. (ii) income above and below the 15th percentile for income. About 50% of cases and 50% of controls had missing data for this variable. (iii) Head of household education: binary variable, either less than or more than 4 years of education.

### Household characteristics

(i) having piped water inside the house; (ii) number of goods in each household. Goods included radios, refrigerators, televisions, washing machines, microwaves, telephones, computers, cars and air conditioners. The minimum number of goods was 0 and the maximum number of goods for a household was 10. The final variable used in the study was binary: households containing 0 to 1 goods and those who had 2 or more goods.

### Analysis

The distribution of each of the potential risk factors and age (the matched variable) among the cases and controls was reported in Table 1 Matched analysis was performed using conditional logistic regression. Univariate conditional logistic regression was performed to obtain crude odds ratios, 95% confidence intervals and likelihood ratio test p-values (Table 2). A backward selection technique was performed to obtain a final multivariate model which is shown in Table 3. Cigarette smoking was left in the final model because it was an original hypothesis. In order to explore which variables were responsible for confounding the crude relationship between smoking and tuberculosis, bivariate and multivariate adjusted odds ratio for smoking and tuberculosis were analyzed (Table 4). Population attributable fractions (PAFs) or proportion of all cases in the whole study population that may be attributed to the exposure, as PAF = P_cases_((RR-1)/RR), where P_cases_ is the proportion of cases were exposed to the risk factor and RR is the adjusted relative risk, here approximated by the odds ratio (OR). Analysis was conducted using STATA 9.2 (Stata Corp, College Station, TX, USA).

**Table 1:** **Proportion of cases and controls characteristics and risk factors for tuberculosis**

		**Cases**		**Controls**	
		**(n = 169)**		**(n = 477)**	
**Variable**		**No**	**%**	**No**	**%**
***Biological factors***					
Age (Matched)	7 to 9	17	10	48	10
	10 to 14	54	32	152	32
	15 to 19	98	58	277	58
Sex	Male	99	59	209	44
	Female	70	41	268	56
Alcohol	No	136	80	380	79.7
	Yes	33	20	94	19.7
	Missing data	0	0	3	0.6
Smoking	No	152	90	454	95.2
	Yes	17	10	22	4.6
	Missing data	0	0	1	0.2
***Contact history***					
Contact history	No contact	97	57	421	89
	Sleep house	44	26	11	2
	Not sleep house	28	17	45	9
***Socio-economic factors***					
Relation to HH	1st degree*	135	80	432	91
	2nd degree**	32	19	40	8
	Missing data	2	1	5	1
Born district	Yes	148	88	431	90.4
	No	21	12	45	9.4
	Missing data	0	0	1	0.2
Literacy	Yes	156	92	468	98
	No	13	8	9	2
Information on HH***					
HH pay	Yes	106	63	315	66
	No	63	37	161	33.8
	Missing data	0	0	1	0.2
Income of HH	0-15th percentile	16	10	9	1.9
	>15th percentile	73	43	154	32.3
	Missing data	80	47	314	65.8
HH Education	<4 years	77	46	226	47.4
	>4 years	92	54	234	49.1
	Missing data	0	0	17	3.6
***HH Characteristics***					
Piped water	Yes	150	89	467	97.9
	No	19	11	7	1.5
	Missing data	0	0	3	0.6
Goods	0 to 1	7	4	12	2.5
	≥2	160	95	454	95.2
	Missing data	2	1	11	2.3

Recife, Brazil 2001–2005.

*1st degree refers to sons or daughters or grandsons/granddaughters.

**2nd degree refers to other relatives or those not related.

***HH is head of the household.

**Table 2:** **Crude matched adjusted ORs of risk factors for tuberculosis**

	**Crude OR**	**95%** **CI**	**LRT p-value**
**Variable**			
***Biological factors***			
Sex			0.0009
Female	Reference	-	
Male	1.82	1.28 to 2.60	
Alcohol			0.9568
No	Reference	-	
Yes	0.99	.60 to 1.61	
Smoking			0.0089
No	Reference	-	
Yes	2.66	1.28 to 5.52	
***Contact history***			
Contact history			<.0001
No contact	Reference	-	
Sleep same house	27.72	10.79 to 71.21	
Not sleep same house	3.21	1.86 to 5.55	
***Socioeconomic variables***			
Relation to HH			0.0012
1st degree	Reference	-	
2nd degree	2.64	1.57 to 4.42	
Born district			0.2524
Yes	Reference	-	
No	1.43	.78 to 2.60	
Literacy			0.0009
Yes	Reference	-	
No	4.42	1.82 to 10.76	
Variable	Crude OR	95% CI	LRT p-value
***Information on HH***			
HH pay			0.4474
Yes	Reference	-	
No	1.16	.79 to 1.72	
Income of HH*			.0137
>15th percentile	Reference	-	
0-15th percentile	4.15	1.25 to 13.75	
HH Education			0.4880
>4 years	Reference	-	
<4 years	0.88	.60 to 1.27	
***Household Char.***			
Piped water			<.00001
Yes	Reference	-	
No	10.17	3.77 to 27.48	
Goods			0.3568
0 to 1	Reference	-	
≥2	0.62	.23 to 1.68	

*Crude ORs estimated by univariate conditional logistic regression.

**Table 3:** **Final model of risk factors for tuberculosis**

**Variable**		Adjusted OR*	**95%** **CI**	**LRT p-value**	**PAF (%)**
Sex				0.0115	
	Female	-			
	Male	1.77	1.13 to 2.77		26
Contact history				<0.00001	
	No contact	-			
	Sleep same house	32.05	10.92 to 94.05		25
	Not sleep same house	3.97	2.16 to 7.31		13
Relation to HH				0.0031	
	1st degree	-			
	2nd degree	2.67	1.40 to 5.08		12
Literacy				0.0343	
	Yes	-			
	No	3.62	1.08 to 12.07		6
Piped water				0.0004	
	Yes	-			
	No	7.35	2.27 to 23.81		10
Smoking					
	No			0.3415	4
	Yes	1.58	0.62 to 4.02		

Recife, Brazil, 2001–2005.

*ORs are adjusted for all other variables in the table.

**Table 4:** **Confounders of the relationship between smoking and TB**

**Variable**	**OR (smoking on TB)**	**95%** **CI**
**Crude**	2.66	1.28 to 5.52
Multivariate*	1.58	.62 to 4.02
Sex	2.19	1.19 to 2.46
Contact	2.26	1.00 to 5.13
Relation to HH	2.68	1.24 to 5.78
Literacy	2.49	1.19 to 5.21
Piped water	2.21	1.02 to 4.76
Sex and contact	1.95	.85 to 4.49
Sex and piped water	1.87	.86 to 4.05
Contact and piped water	1.80	.75 to 4.32
Sex and contact and piped water	1.61	.67 to 3.87
**Sex, contact, piped water, literacy**	1.58	.65 to 3.83

*Multivariate OR is adjusted for sex, contact history, relation to HH, literacy, piped water.

Ethical approval was granted by the Ethical Committee of the Universidade Federal do Pernambuco. Written signed consent was received for all participants.

## Results

Analysis was completed on 169 cases and 477 matched controls. The age of the subjects ranged from 7 to 19. The mean age for cases and controls was 14.4 years. More cases were male (58%). The distribution of characteristics and risk factors among the cases and controls is presented in Table 1. Approximately 10% of males are smokers compared to only 3% of females; all adolescents who reported smoking were aged 15 to 19 years old while 2 of a total of 127 adolescents who reported alcohol consumption were aged 10 to 14 years and 125 were aged 15 to 19 years (data not shown). Crude, matched odds ratios of risk factors are presented in Table 2. The strongest variable associated with TB was sleeping in the same house with someone with TB (OR = 27.72 95% CI 10.79, 71.21). Living in a house with no piped water was also strongly associated (OR = 10.17 95% CI 3.77, 27.48). Other factors statistically significantly associated with tuberculosis were male sex (OR = 1.82 95% CI 1.28, 2.60), cigarette smoking (OR = 2.66 95% CI 1.28, 5.52) and relationship to head of household (OR = 2.64 95% CI 1.57, 4.42), illiteracy (OR = 4.42 95% CI 1.82, 10.76) and low income (OR = 3.94 95% CI 1.56, 9.97). There was very weak evidence that alcohol consumption, place of birth, Head of household being in paid employment, education of head of the household and the number of goods owned by each household were risk factors for tuberculosis in this age group.

In the final model (Table 3) using a multivariable conditional logistic regression, the association between cigarette smoking and tuberculosis became weaker (OR = 1.58, 95%CI 0.62 to 4.02; PAF =4%) and was no longer statistically significant. History of contact with a case of tuberculosis (in the same house, OR = 32.05 95% CI 10.92, 94.05; not in the same house, OR = 3.97 95% CI 2.16 to 7.31) and living in a house with piped water (OR = 7.35 95% CI 2.27, 23.81) had the strongest associations with tuberculosis disease. When we calculated the population attributable fraction, contact history explained 38% and piped water explained 10% of all the tuberculosis cases in the population. There was evidence for associations with male sex (OR = 1.77 95% CI 1.13, 2.77; PAF = 26%), illiteracy (OR = 3.62 95% CI 1.08, 12.07: PAF 6%) and relationship to head of house (OR = 2.67 95% CI 1.40, 5.08: PAF = 12). No single risk factor was responsible for the loss of significance in the association between smoking and tuberculosis. Only when at least two additional variables were included in the model did smoking loose its significance, but the OR remained of similar magnitude 1.6 (Table 4).

Effect of being male was similar in those aged 7–14 (crude OR = 1.44 95% CI .82, 2.54), and in adolescents (aged 15–19) (crude OR = 1.39 95% CI 1.39, 3.51) but this was only statistically significant in the older age groups (data not shown).

## Discussion

Contact with a case of tuberculosis was the most important risk factor for tuberculosis, accounting for 37% of the cases (25% from household contacts and 12% from other contacts). Living in a household and not being related to the head of the household contributed 12% of cases. Living in a household with no piped water contributed 10% and illiteracy 6%. The risk associated with living in a household with no piped water was much higher for males than for females. Smoking explained 4% of cases. Traditional socio economic factors, like household income and ownership of goods were not significantly associated.

Contact history, as well as closeness of contact, is well-defined as a risk factor for tuberculosis [18-22]. It is of particular interest that such a high proportion of cases (25%) were attributed to household contact in our study. Most other studies show slightly lower percentages: 12% in a multicentre study from 3 West African countries [20] and from Malawi [21], 23% from Liverpool [22] and a surprisingly high 40% from the Gambia [18].

As contact with a tuberculosis patient was reported by the child/adolescent or their parents/guardians, it may have occurred that cases or their parents/guardians were more likely to remember a contact outside of the household; however it would not be expected that it would also happen in relation to close contacts.

Socio economic status is traditionally associated with tuberculosis [23-26]. In this study, only illiteracy, living in a house with no piped water and living in a household but not being a first degree relative of the head of the family were associated with tuberculosis. We do not interpret the increased risk in those living in a household with no piped as causal but as a proxy for extremely poor living conditions. Individuals who were not first degree relatives to the head of the household where they lived had an increased risk of tuberculosis. In this society and age group, children and adolescents living in a household where they are not part of the nuclear family would tend to be poorer relations, and may also not receive as much resources or support than the closer relatives; in the older age group they might be servants. Illiteracy in children aged 7 or more and adolescents may express the lack of opportunity to attend school and the socioeconomic conditions behind it, being a proxy of low standard of living. There may have been some degree of misclassification in relation to the response to this question but there is no reason why it should differ between cases and controls. Thus the association found may have been underestimated. In another study of our group [26] in which a multilevel analysis was performed, an association between tuberculosis and illiteracy was found at the individual as well as at the area level. Other measures of poverty, traditionally associated with tuberculosis in adults were not significant. It appears that the measures of socio economic status related to tuberculosis in this age group and setting tend to relate to housing conditions and relative position in the family more than to income and property.

Incidence of tuberculosis are higher in males worldwide. Possible reasons for the a higher rate of tuberculosis in males include differences in immunity [6] more frequent external contacts for young men than young women [7] and differences in health seeking behavior [6,7]. The latter is not likely to be the reason for the increase in risk in men as in Brazil there is universal access to tuberculosis diagnosis, health care use is similar for boys and girls until age 15, and higher for women after age 15 [27], rates of completing secondary school are similar for men and women and almost all schools are mixed. However, it is possible that in the work place rates of contact are different between man and women.

Smoking, a significant risk factor in the univariate analysis, was not significantly associated in the multivaried analysis although the increase in risk was still of about 50%. In this study, smokers were more likely to be male and not have access to piped water than non-smokers, and these factors explained a proportion of the association between smoking and the risk of TB. The most dominant biological reason for increased susceptibility to TB is that smoking leads to down-regulation of macrophage TNF-α in the lungs, rendering the patient more susceptible to the development of TB disease [14]. A study in India found a strong dose–response relationship between tobacco smoking and pulmonary tuberculosis [12]. Previous studies have also found that both passive and active exposure to tobacco smoke have been shown to be associated with TB disease [13]. Furthermore, a recent meta-analyses provided evidence that smoking is a risk factor for TB infection and disease [11]. The prevalence of smoking was low: 39 children, 6% of the population, all over aged 15 years, were labeled as smokers. It remains a possibility that the lack of significant association between smoking and tuberculosis in this study was due to lack of power given the small number of reported smokers. Though small to reach statistical power, the rate smoking reported, as well as of alcohol use, was not negligible for a country like Brazil where there are laws prohibiting the sale to minors of products containing ingredients that can cause physical or mental addiction.

There are a number of limitations to this study. In case control studies there is always a possibility of overmatching. Controls from the same neighborhoods could have been more homogeneous with regards to smoking than the general population. The power may have been insufficient to provide definitive evidence of the effect of smoking and to study differences among adolescents and older children. It may have happened that individuals interviewed in the clinics (cases or their parents/guardians) reported better living conditions than those interviewed in their homes (controls or their parents/guardians), which may have led to an underestimation of the association with the socioeconomic variables. Social overcrowding was not analyzed in this study but it is likely to be related to other socioeconomic variables considered.

## Conclusion

In conclusion, it seems that close contact with a case of tuberculosis is responsible for the largest proportion of cases in this age group. Social factors tend to be those related to household rather than income among children in the study. Smoking was associated, but possibly given the small numbers, this was not statistically significant. We recommend further studies to explore the relationship of cigarette smoking and tuberculosis by using a larger sample size and a more accurate measure on the number of cigarettes smoked. It may be of interest to study separate risk factors for adolescents as compared to young children. Future studies should also explore whether the effect of social factors, in particular bad living conditions, are more marked in males than in females.

## References

[1] World Health Organization: **Global Tuberculosis Report 2013.** http://www.who.int/iris/handle/10665/91355#sthash.jqI0CUqx.dpuf

[2] World Health Organization. **Global Tuberculosis Report 2014.** http://apps.who.int/iris/bitstream/10665/137094/1/9789241564809_eng.pdf?ua=1

[3] Meya DB, McAdam KP (2007). The TB pandemic: an old problem seeking new solutions. J Intern Med.

[4] Giacchino R, di Martino L, Losurdo G, Pisanti A (2003). Tuberculosis infection and disease in immigrant children. Infez Med.

[5] Che D, Antoine D (2009). Immigrants et tuberculose: données épidémiologiques récentes. Med Mal Infect.

[6] Watkins RE, Plant AJ (2006). Does smoking explain sex differences in the global tuberculosis epidemic?. Epidemiol Infect.

[7] Holmes CB, Hausler H, Nunn P (1998). A review of sex differences in the epidemiology of tuberculosis. Int J Tuberc Lung Dis.

[8] Fox GJ, Barry SE, Britton WJ, Marks GB (2013). Contact investigation for tuberculosis: a systematic review and meta-analysis. Eur Respir J.

[9] Jonsson J, Kan B, Berggren I, Bruchfeld J (2013). Extensive nosocomial transmission of tuberculosis in a low-incidence country. J Hosp Infect.

[10] Milburn H (2007). Key issues in the diagnosis and management of tuberculosis. J R Soc Med.

[11] Bates MN, Khalakdina A, Pai M, Chang L, Lessa F, Smith KR (2007). Risk of tuberculosis from exposure to tobacco smoke: a systematic review and meta-analysis. Arch Intern Med.

[12] Kolappan C, Gopi PG (2002). Tobacco smoking and pulmonary tuberculosis. Thorax.

[13] Chiang CY, Slama K, Enarson DA (2007). Associations between tobacco and tuberculosis. Int J Tuberc Lung Dis.

[14] Davies PD, Yew WW, Ganguly D, Davidow AL, Reichman LB, Dheda K, Rook GA (2006). Smoking and tuberculosis: the epidemiological association and immunopathogenesis. Trans R Soc Trop Med Hyg.

[15] Nelson LJ, Wells CD (2004). Global epidemiology of childhood tuberculosis. Int J Tuberc Lung Dis.

[16] Ministerio da Saude. Sistema de Informação de Agravos de Notificação – Sinan. Available at: http://dtr2004.saude.gov.br/sinanweb/tabnet/dh?sinannet/tuberculose/bases/tubercbrnet.def.

[17] Dantas OM, Ximenes RA, de Albuquerque MF, da Silva NL, Montarroyos UR, de Souza WV, Pereira TC, Campelo AR, Rodrigues LC (2006). A case–control study of protection against tuberculosis by BCG revaccination in Recife. Brazil Int J Tuberc Lung Dis.

[18] Hill PC, Jackson-Sillah D, Donkor SA, Otu J, Adegbola RA, Lienhardt C (2006). Risk factors for pulmonary tuberculosis: a clinic-based case control study in The Gambia. BMC Public Health.

[19] Sinfield R, Nyirenda M, Haves S, Molyneux EM, Graham SM (2006). Risk factors for TB infection and disease in young childhood contacts in Malawi. Ann Trop Paediatr.

[20] Lienhardt C, Fielding K, Sillah JS, Bahb B, Gustafson P, Warndorff D, Palayew M, Lisse I, Donkor S, Diallo S, Manneh K, Adegbola R, Aaby P, Bah-Sow O, Bennett S, McAdam K (2005). Investigation of the risk factors for tuberculosis: a case–control study in three countries in West Africa. Int J Epidemiol.

[21] Crampin AC, Floyd S, Ngwira BM, Mwinuka V, Mwaungulu JN, Branson K, Fine PEM, Glynn JR (2008). Assessment and evaluation of contact as a risk factor for tuberculosis in rural Africa. Int J Tuberc Lung Dis.

[22] Tocque K, Bellis MA, Beeching NJ, Syed Q, Remmington T, Davies PDO (2001). A case–control study of lifestyle risk factors associated with tuberculosis in Liverpool, Noerth-West England. Eur Respir J.

[23] Lienhardt C (2001). From exposure to disease: the role of environmental factors in susceptibility to and development of tuberculosis. Eepidemiol Rev.

[24] Souza WV, Ximenes R, Albuquerque MF, Lapa TM, Portugal JL, Lima MLC, Martelli CMT (2000). The use of socioeconomic factors in mapping tuberculosis risk areas in a city of northeastern Brazil. Pan Am J Public Health.

[25] Russell S (2004). The economic burden of illness for households in developing countries: a review of studies focusing on malaria, tuberculosis, and human immunodeficiency virus/acquired immunodeficiency syndrome. Am J Trop Med Hyg.

[26] Ximenes RAA, Albuquerque MFPM, Souza WV, Montarroyos UR, Diniz GT, Luna CF, Rodrigues LC (2009). Is it better to be rich in a poor area or poor in a rich area? A multilevel analysis of a case–control study of social determinants of tuberculosis. Int J Epidemiol.

[27] Travassos C, Vaiacava F, Pinheiro R (2002). Utilização dos serviços de saúde no Brasil: gênero, características familiares e condição social. Pan Am J Public Health.

